# Technical Note: Volumetric computed tomography for radiotherapy simulation and treatment planning

**DOI:** 10.1002/acm2.13336

**Published:** 2021-07-09

**Authors:** Heather M. Young, Claire Keun Sun Park, Oi‐Wai Chau, Ting‐Yim Lee, Stewart Gaede

**Affiliations:** ^1^ Department of Medical Biophysics University of Western Ontario London Canada; ^2^ London Regional Cancer Program London Canada; ^3^ Robarts Research Institute University of Western Ontario London Canada; ^4^ Lawson Health Research Institute London Canada

**Keywords:** radiation treatment planning, radiotherapy simulation, volumetric computed tomography

## Abstract

**Purpose:**

For lung and liver tumors requiring radiotherapy, motion artifacts are common in 4D‐CT images due to the small axial field‐of‐view (aFOV) of conventional CT scanners. This may negatively impact contouring and dose calculation accuracy and could lead to a geographic miss during treatment. Recent advancements in volumetric CT (vCT) enable an aFOV up to 160 mm in a single rotation, which may reduce motion artifacts. However, the impact of large aFOV on CT number required for dose calculation needs to be evaluated before clinical implementation. The objective of this study was to determine the utility of a 256‐slice vCT scanner for 4D‐CT simulation by evaluating image quality and generating relative electron density (RED) curves.

**Methods:**

Images were acquired on a 256‐slice GE Revolution CT scanner with 40 mm, 80 mm, 120 mm, 140 mm, and 160 mm aFOV. Image quality was assessed by evaluating CT number linearity, uniformity, noise, and low‐contrast resolution. The relationship between each quality metric and aFOV was assessed.

**Results:**

CT number linearity, uniformity, noise, and low‐contrast resolution were within the expected range for each image set, except CT number in Teflon and Delrin, which were underestimated. Spearman correlation coefficient (ρ) showed that the CT number for Teflon (ρ = 1.0, *p *= 0.02), Delrin (ρ = 1.0, *p *= 0.02), and air (ρ = 1.0, *p *= 0.02) was significantly related to aFOV, while all other measurements were not. The measured deviations from expected values were not clinically significant.

**Conclusion:**

These results suggest that vCT can be used for CT simulation for radiation treatment planning.

## 1 INTRODUCTION

Four‐dimensional computed tomography (4D‐CT) is part of the standard of care for radiotherapy treatment planning for tumors influenced by respiratory motion, such as in lung and liver cancer. Lung and liver tumors have been shown to move up to 50 mm during free breathing.^1^, ^2^ However, conventional CT scanners have a narrow axial field‐of‐view (aFOV) of 40 mm or less, so images must be acquired at multiple couch positions and recombined to construct a complete image set. Irregular breathing motion during this acquisition can result in significant motion‐induced artifacts.^3^ These artifacts may lead to inaccuracies in target delineation and contouring organs at risk due to geometric distortion,^4^, ^5^ and have been shown to significantly impact clinical outcomes in patients with lung and liver lesions.^6^

New volumetric CT scanners (also called area detector scanners) with 160 mm aFOV greatly reduce the artifacts caused by irregular breathing motion during imaging.^7^ However, accurate CT density measurements are critical for radiation dose calculations, and the volumetric geometry used in vCT may result in cone‐beam artifacts which could distort the measured CT number. This effect could limit the application of vCT for radiation treatment planning and dose calculations. Previous studies have investigated image quality metrics in other scanners, thus finding evidence of cone‐beam artifacts that were not clinically significant.^8^ In addition, one recent study investigated dose and effective energy in a 160mm aFOV scan in a volumetric scanner.^9^

The objective of this study is to examine the image quality of vCT images acquired on the GE Revolution CT scanner across multiple axial fields‐of‐view using a Catphan 504 phantom (The Phantom Laboratory Inc., Greenwich, USA). CT number linearity, uniformity, noise, and low‐contrast resolution were assessed across increasing aFOV. Furthermore, we generated relative electron density (RED) curves for clinical implementation in radiotherapy treatment planning systems.

## 2 MATERIALS AND METHODS

### 2.1 Image acquisition

The Catphan 504 phantom was imaged on a 256‐slice GE Revolution volumetric CT scanner (GE Medical Systems, Madison, USA) at 120 kV, 590 mA, 0.625 mm pixel spacing, and 1.0 s rotation speed with 980 projections. Images were reconstructed with the “Standard” convolution kernel and the filter type “Medium Filter,” with a cut‐off frequency (5% MTF) of 8 lp/cm. Repeated scans with aFOV of 40, 80, 120, 140, and 160 mm were performed and the entire phantom was scanned in the axial mode for each aFOV. Phantom specifications for expected CT number and relative electron density (RED) for the phantom density inserts were taken from the Catphan 504 manual,^10^ as summarized in Table 1. To test for changes in image uniformity along the z‐axis, the Catphan was imaged with an aFOV of 160 mm positioned in the center of the bore and at the outer edges of the scanner.

**TABLE 1:** Mean CT number measured in circular regions in the Catphan 504 phantom density inserts. Expected CT number given as [minimum, maximum] and relative electron density (RED) given in the Catphan manual

Material	Measured CT Number [HU]					Expected CT Number [HU]	RED
	40 mm	80 mm	120 mm	140 mm	160 mm		
Teflon	889.01	890.37	900.94	904.68	904.86	[941, 1060]	1.868
Delrin	302.44	306.08	311.66	316.84	317.18	[344, 387]	1.363
Acrylic	121.68	120.89	120.48	121.21	120.13	[92, 137]	1.147
Polystyrene	−36.40	−36.30	−36.30	−35.80	−37.20	[−65, −29]	0.998
LDPE	−94.41	−92.85	−94.40	−93.15	−93.10	[−121, −87]	0.945
PMP	−187.45	−185.72	−185.8	−185.21	−185.61	[−220, −176]	0.853
Air	−995.73	−992.53	−992.24	−992.2	−990.92	[−1046, −986]	0.001

Abbreviation: LDPE; Low‐density polyethylene; PMP; polymethylpentene; RED, relative electron density.

### 2.2 Image analysis

Image analysis for CT number measurements including linearity, uniformity, noise, and low‐contrast resolution was performed using ITK‐SNAP (version 3.4.0)^11^ for all aFOV values.

#### 2.2.1 Linearity

**FIGURE 1:**
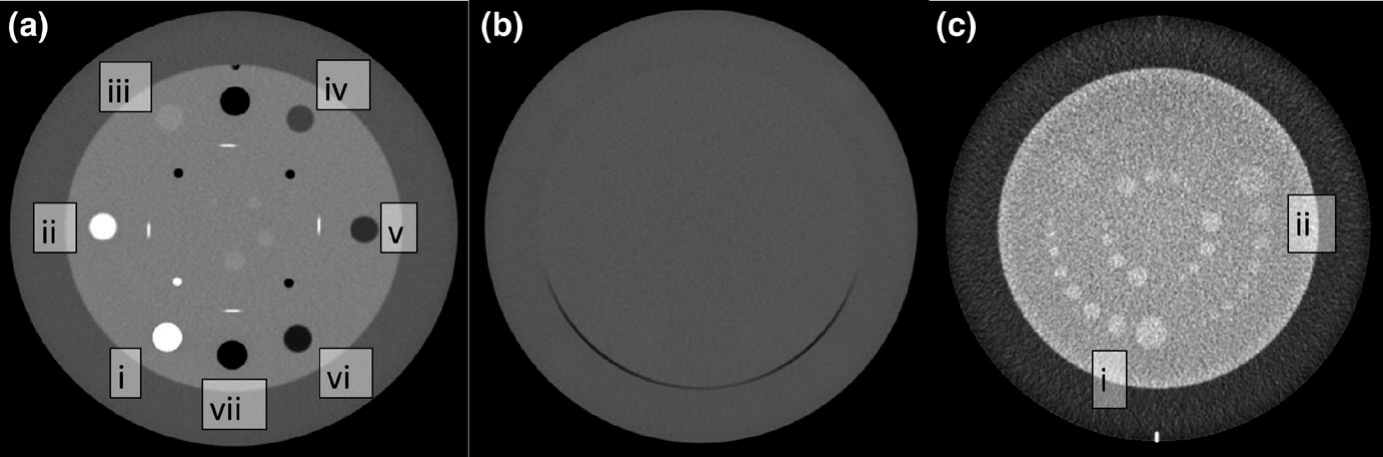
Axial CT images of Catphan 504 phantom modules analyzed using ITK‐Snap. (A) The sensitometry module used for linearity measurements containing (i) Teflon, (ii) Delrin, (iii) acrylic, (iv) polystyrene, (v) LDPE, (vi) PMP, and (vii) air density inserts (window width/window level = 600/0 HU). (B) The uniformity (CTP486) module used to measure uniformity and noise (window width/window level = 600/0 HU). (C) The low‐contrast resolution (CTP515) module used to measure contrast‐to‐noise (CNR) in the (i) 1.0% and (ii) 0.5% nominal contrast level targets (window width/window level = 120/20 HU)

Linearity was measured by measuring the average CT number in a region of interest (ROI) within the sensitometry (CTP404) module in inserts containing Teflon, Delrin, acrylic, polystyrene, low‐density polyethylene (LDPE), polymethylpentene (PMP), and air as shown in Figure 1. These values were compared to the expected range given in the Catphan manual.

#### 2.2.2 CT number uniformity

CT number uniformity across an image was evaluated using the phantom uniformity (CTP486) module using circular ROIs (area = 520 mm^2^) manually placed in the center of the phantom (1 ROI) and at the periphery of the phantom (4 ROIs) as described in the phantom manual. The mean intensity was measured in each ROI and the absolute difference between the center region and maximum periphery region was calculated using equation 1:

(1) $$Max\;difference=\left|{\mathrm{CT}}_{mean,\;\;\;center}‐{\mathrm{CT}}_{max,\;\;\;periphery}\right|$$

The CT number [HU] difference measured in the uniformity module is expected to be within 2% (20 HU) of that of water, usually in the range 5–18 HU.^10^

To test image uniformity in the z‐direction, horizontal image profiles were taken across a uniform region of the phantom positioned at the outer edges of the scanner bore and in the center. These profiles were visually examined for artifacts. A longitudinal profile was also taken in a uniform region of the phantom to evaluate possible correlations between CT number and position on the z‐axis.

#### 2.2.3 Image noise

Image noise was quantified by measuring the standard deviation of the CT number within a circular ROI (area = 2500 mm^2^) in the uniformity module.

#### 2.2.4 Low‐contrast resolution

Low‐contrast resolution was assessed using three nominal target contrast levels of 1.0%, 0.5%, and 0.3%. For each level, an image from the low‐contrast (CTP515) module was displayed with fixed image settings (window = 400, level = 40). Three independent observers manually counted the number of targets visible in the image. The viewing conditions including the computer screen and location brightness, and physical environmental surroundings were kept constant.

#### 2.2.5 Contrast‐to‐noise ratio (CNR)

To measure CNR, targets were selected in the 1.0% and 0.5% contrast regions in the low‐contrast module and two background targets of the same size (area = 90 mm^2^) were selected adjacent to the targets as shown in Figure 1. CNR was measured according to Equation 2 below:

(2) $$CNR=\frac{2{\left(C_{0}‐C_{b}\right)}^{2}}{\sigma_{0}‐\sigma_{b}}$$

where $C_{0}$ and $\sigma_{0}$ are the mean and standard deviation of the signal from the target and $C_{b}$ and $\sigma_{b}$ are the mean and standard deviation of the signal from the background.

#### 2.2.6 RED calibration curves

RED calibration curves for each aFOV were generated by plotting the measured CT number and the known RED obtained from the Catphan 504 phantom manual as shown in Table 1.

### 2.3 Statistical analysis

All statistical analysis was performed using Prism 7.0 (GraphPad Software Inc., California, USA). Spearman correlation coefficient (ρ) was used to assess correlations between measured parameters and aFOV, and linear regression was used to assess the strength of significant correlations.

## 3 RESULTS

### 3.1 Linearity

The measured CT number for all aFOV was within the range specified in the Catphan 504 manual for air, PMP, LDPE, polystyrene, and acrylic. CT number was underestimated at all aFOV settings for the two high‐density inserts: Delrin and Teflon. All CT number measurements are shown in Table 1.

**FIGURE 2:**
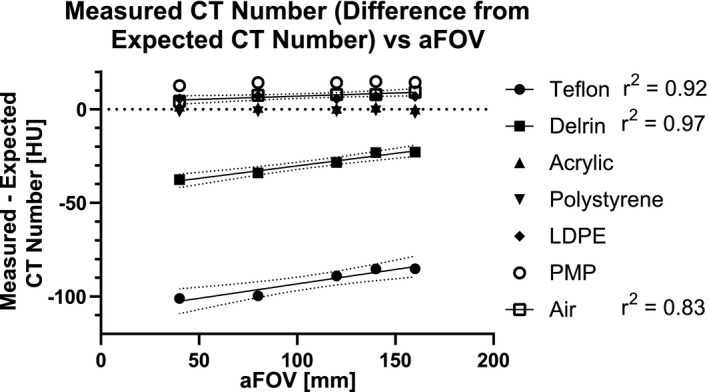
Difference between measured CT number in the sensitometry (CTP404) module and the expected CT number for each aFOV in the vCT images. CT number and aFOV were not significantly correlated for: acrylic (ρ = −0.7, *p *= 0.2), polystyrene (ρ = −0.05, *p* > 0.99), LDPE (ρ = 0.4, *p *= 0.5), and PMP (ρ = 0.8, *p *= 0.1). Measured CT number was significantly correlated with aFOV for: Teflon (ρ = 1.0, *p *= 0.02, *r* ^2^ = 0.92), Delrin (ρ = 1.0, *p *= 0.02, r^2^ = 0.97), and air (ρ = 1.0, *p *= 0.02, *r* ^2^ = 0.83)

Measured CT number was significantly correlated with aFOV in Teflon (ρ = 1.0, *p *= 0.02, *r* ^2^ = 0.92), Delrin (ρ = 1.0, *p *= 0.02, *r* ^2^ = 0.97), and air (ρ = 1.0, *p *= 0.02, *r* ^2^ = 0.83) with a significantly non‐zero slope (Teflon: *p *= 0.01, Delrin: *p *= 0.002, air: *p *= 0.03) as shown in Figure 2. Measured HU and aFOV were not significantly related for acrylic (ρ = −0.7, *p *= 0.2), polystyrene (ρ = −0.05, *p *= 0.9), LDPE (ρ = 0.4, *p *= 0.5), or PMP (ρ = 0.8, *p *= 0.1).

### 3.2 Uniformity

**FIGURE 3:**
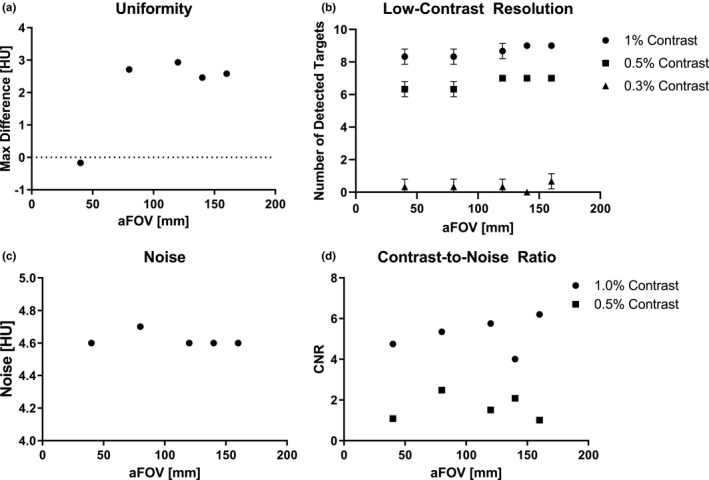
(A) Maximum absolute difference [HU] between central and peripheral ROIs versus axial‐field‐of‐view (aFOV) in the uniformity (CTP486) module (ρ = 0.20, *p *= 0.78). (B) Mean number of observed targets counted by three independent observers versus aFOV in the low‐contrast (CTP515) module at contrast levels: 1.0% (ρ = 0.95, *p *= 0.067), 0.5% (ρ = 0.87, *p *= 0.20), and 0.3% (ρ = 0.22, *p* > 0.99). (C) Noise in the uniformity ROI versus aFOV in the uniformity (CTP486) module (ρ = −0.35, *p *= 0.40). (D) Contrast‐to‐noise ratios (CNR) in nominal contrast levels 1.0% and 0.5% versus aFOV in the low‐contrast (CTP515) module (ρ = 0.40, *p *= 0.52 (1.0%), ρ = −0.30, *p *= 0.68 (0.5%))

The average CT number in each uniformity ROI across all aFOV images was (10.8 +/− 1.2) HU (A), (11.3 +/− 0.7) HU (B), (9.9 +/− 0.4) HU (C), (8.6 +/− 0.6) HU (D), and (9.9 +/− 0.5) HU (E). The mean measured CT number in all ROIs was within 2% (20 HU) of the expected value (0 HU). The maximum absolute difference in CT number between central and peripheral ROIs was (2.1 +/− 1.1) HU and <5 HU for all measurements. The maximum absolute difference in CT number was not significantly correlated with aFOV (*ρ* = 0.20, *p* = 0.78) as shown in Figure 3.

### 3.3 Noise

Noise in the uniformity module for all aFOV values was 4.6 +/− 0.04 HU. Noise was not significantly correlated with aFOV (ρ = −0.4, *p *= 0.4) as shown in Figure 3.

### 3.4 Low‐contrast resolution

Three independent observers counted the number of targets in the low‐contrast resolution module at contrast levels 1.0%, 0.5%, and 0.3% for each aFOV. The number of observed targets was 8.7 +/− 0.5 at 1.0%, 6.7 +/− 0.4 at 0.5%, and 0.33 +/− 0.4 at 0.3% as shown in Figure 3. The number of observed targets was not significantly correlated with aFOV at 1.0% (*p *= 0.067), 0.5% (*p *= 0.2), or 0.3% (*p* > 0.99).

Contrast‐to‐noise ratio (CNR) was analyzed on the low‐contrast resolution image (Figure 1) using the 15 mm targets in the 1.0% and 0.5% nominal contrast levels. The CNR at 1.0% was 5.2 +/− 0.9 and at 0.5% was 1.6 +/− 0.6. CNR was not significantly related to aFOV at 1.0% (ρ = 0.4, *p *= 0.5) or 0.5% (ρ = −0.3, *p *= 0.7) as shown in Figure 3.

### 3.5 Longitudinal directional dependence

**FIGURE 4:**
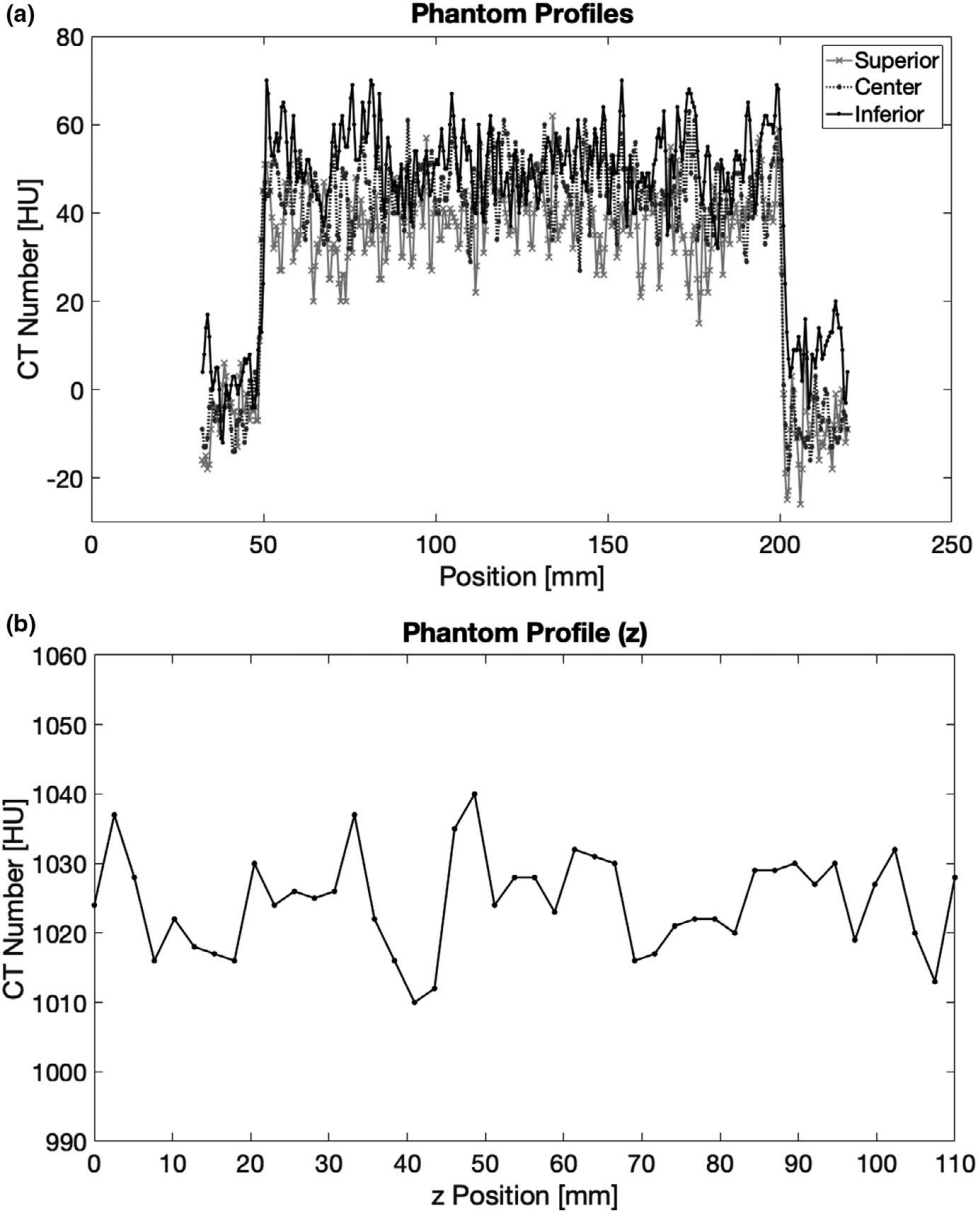
(A) Horizontal profiles of a uniform volume in the Catphan 504 phantom taken when the phantom was in a superior, center, and inferior position along the z‐axis. These profiles appear to be consistent across the imaging volume. (B) A longitudinal profile of the uniform acrylic perimeter of the Catphan phantom. There is no significant relationship between measured CT number and position within the imaging bore (ρ = 0.04, *p *= 0.8)

**FIGURE 5:**
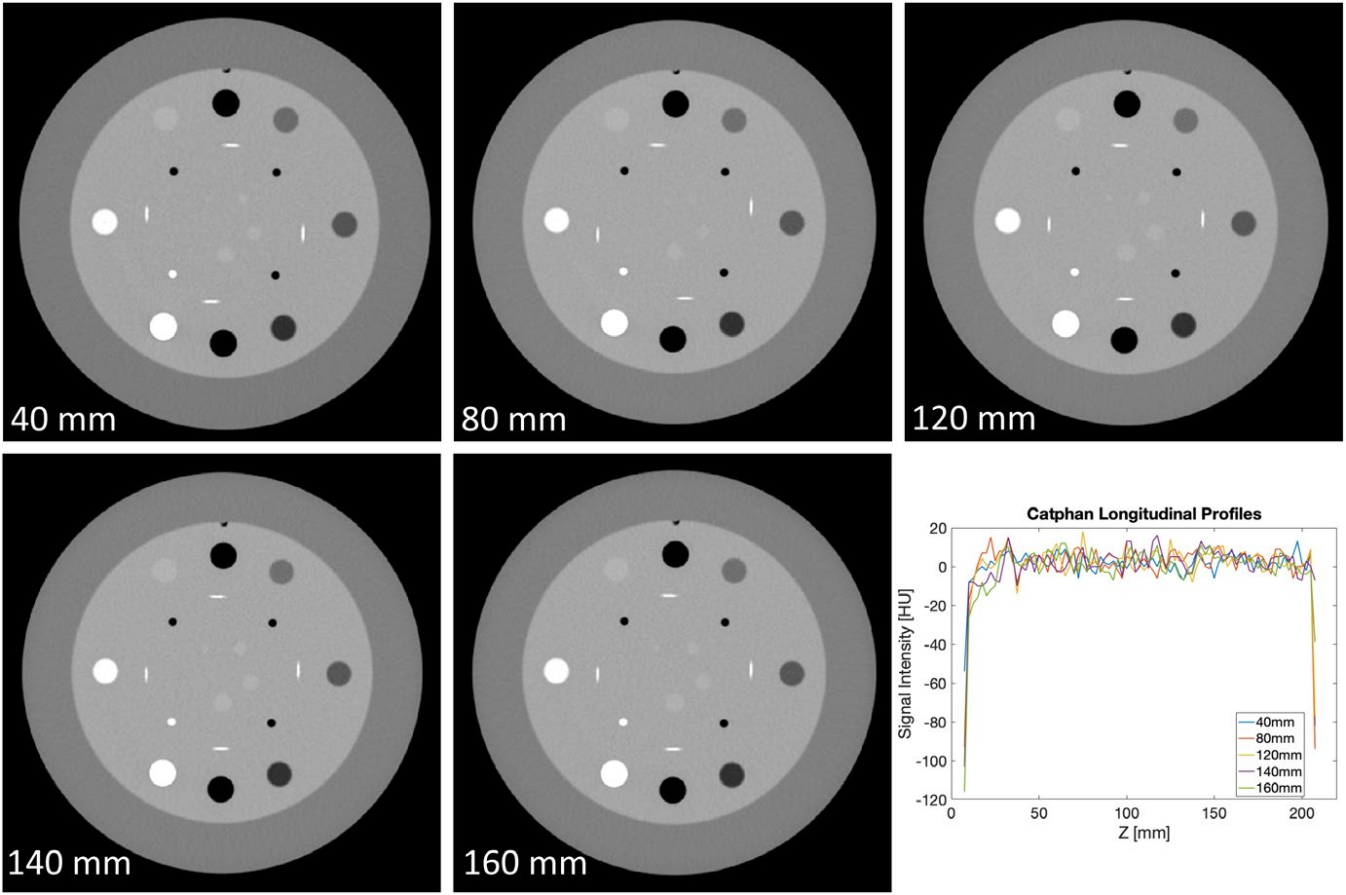
An axial slice of the linearity module in the Catphan 504 phantom acquired at each aFOV value tested in this study, and longitudinal profiles from each image. Visual comparison shows no obvious differences between the images

Horizontal profiles were compared from images taken at the center of the imaging bore of the scanner and at the outer edges of the bore at 160mm aFOV. The profiles were plotted as shown in Figure 4. No visible artifacts were identified and there were no visible differences between the profiles. In the longitudinal profile, as shown in Figure 4, there was no significant correlation between z position and measured CT number (ρ = 0.04, *p *= 0.8). Slices from the linearity module acquired at each aFOV value were also compared by visual inspection and by comparing line profiles through each image as shown in Figure 5. There were no visible differences between the images from each aFOV.

### 3.6 RED calibration

**FIGURE 6:**
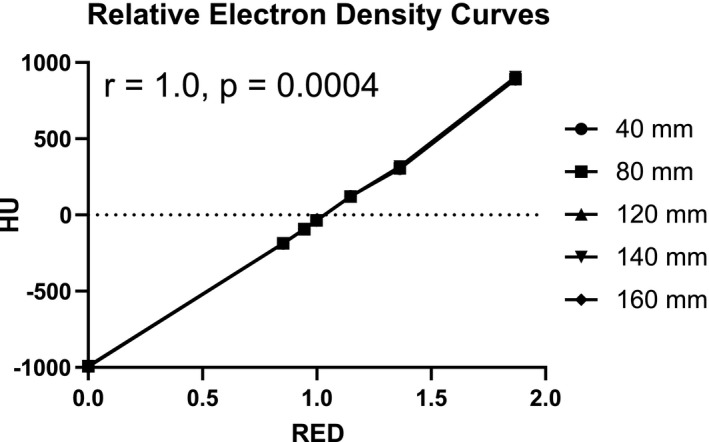
Relative electron density (RED) calibration curves of measured CT number against Catphan 504 Phantom RED specifications for aFOV of 40, 80, 120, 140, and 160 mm on the vCT, and for the clinical CT. All RED curves were highly correlated (ρ = 1.0, *p *= 0.0004)

To perform dose calculations using a CT image, a relative electron density (RED) curve specific to the CT scanner and imaging parameters is required. We generated RED curves for each image set as shown in Figure 6.

## 4 DISCUSSION

In this paper, we assessed the utility of volumetric CT for radiotherapy simulation, treatment planning, and dose calculation. Volumetric CT is particularly useful for dynamic imaging techniques, such as contrast‐enhanced imaging and 4D‐CT, both of which are used in radiation treatment planning. Previous research has investigated the impact of volumetric imaging on these dynamic techniques and has demonstrated that contrast‐enhanced imaging is not negatively affected by cone‐beam artifacts in volumetric imaging^9^, ^12^ and vCT can reduce the presence of respiratory motion artifacts in 4D‐CT used for motion management.^7^ These findings support the potential use of vCT for treatment planning.

We investigated the differences in linearity, RED, noise, and uniformity of images acquired using a 256‐slice vCT scanner at different aFOV settings. The measurements were compared to the Catphan 504 phantom manual to determine if any artifacts were present that could impact the use of this scanner for CT simulation and radiation therapy planning. We also imaged the phantom at different locations along the longitudinal axis of the scanner to test for cone‐beam artifacts.

Our measurements demonstrated that the measured CT number in acrylic, polystyrene, LDPE, PMP, and air was consistent with the values expected from the Catphan manual (Table 1). However, the measured CT number in Teflon and Delrin was underestimated for each axial coverage compared to these specifications. CT density is dependent on various factors including energy, scattering, and reconstruction algorithms.^13^, ^14^ The phantom specifications are defined over 94 different scanners and protocols, and the manual explicitly states that dramatic deviations from the specified HU range are not unusual.^10^ In addition, a single scanner is known to produce variable results over time,^15^ and this variability is expected and accounted for in commissioning and quality assurance protocols. For treatment planning purposes, these variations will be accounted for with the relative electron density curve that is specific to the scanner. Therefore, while these deviations are statistically significant, we do not expect them to be clinically significant. We also observed that the measured CT number in acrylic, polystyrene, LDPE, and PMP was not significantly correlated with aFOV, but Teflon, Delrin, and air increased significantly with aFOV. However, the relative change in CT number was small in all these cases (<5% for Delrin and <1% for all lower density materials). These results suggest that most artifacts of increased aFOV are mitigated by hardware (i.e., an anti‐scatter grid) and algorithms to reduce artifacts in reconstructed images on the CT scanner. In addition, any remaining artifacts are so small that they are not considered clinically significant.

Uniformity measurements were in agreement with the recommended range^16^ for each aFOV measured and were not significantly correlated with aFOV. Noise was also not correlated with aFOV. In our low‐contrast resolution test, there was strong agreement between our observers and the number of targets identified was not significantly related to aFOV. CNR was analyzed for the 1.0% and 0.5% contrast modules and was also not significantly related to aFOV.

This study is limited by the fact that only phantom images were studied. Anthropomorphic phantoms or human images can also be compared between a clinical and volumetric scanner to confirm these findings. In addition, it is noteworthy that the relatively small bore size of this scanner (80 cm) limits its use for radiation therapy planning for some patients. Most clinical CT simulators are wide‐bore scanners to accommodate a range of patient sizes and to include immobilization equipment, which can be very bulky. The small bore of the scanner in this study means that it likely cannot meet all CT simulation needs, as a large bore scanner will be needed for some patients. In the future, large‐bore volumetric CT scanners could enable wider use of this technology in radiation oncology.

These results have significant implications for future clinical research projects. Dynamic contrast‐enhanced CT imaging with a vCT scanner enables high‐resolution functional mapping over a large anatomical volume. This advanced imaging technique can facilitate research projects such as functional avoidance treatment planning or studying imaging biomarkers of treatment response. The use of a vCT scanner for treatment planning facilitates direct access for researchers to volumetric imaging for research studies. Further work must be performed to fully characterize the RED in clinical conditions before this system can be used clinically, in accordance with AAPM recommendations.^16^, ^17^

## 5 CONCLUSIONS

We imaged a Catphan phantom on a vCT scanner to assess image quality and generated RED calibration curves for increasing axial coverage up to 160 mm. CT linearity, uniformity, noise, and low‐contrast resolution were within the expected range for most measurements, and deviations were determined to be not clinically significant. Our results suggest that most artifacts resulting from increased aFOV are mitigated by reconstruction techniques on the scanner, and the remaining artifacts are considered clinically acceptable for radiation treatment planning. This study demonstrates that the vCT scanner can be used for CT simulation and radiotherapy treatment planning.

## CONFLICT OF INTEREST

The authors have no relevant conflicts of interest to disclose.

## AUTHOR CONTRIBUTION

Heather M Young contributed to the data acquisition and analysis and interpretation of the data for this work, drafted the manuscript, and approved the final version. Claire Keun Sun Park contributed to the data acquisition and analysis, contributed to the writing of the manuscript, and approved the final version. Oi‐Wai Chau contributed to data acquisition and writing of the manuscript and approved the final version. Ting‐Yim Lee contributed to data analysis and interpretation, contributed to the drafting of this manuscript, and approved the final version. Stewart Gaede contributed to data acquisition, analysis, and interpretation, contributed to the drafting of this manuscript, and approved the final version.

## Data Availability

The data that support the findings of this study are available from the corresponding author upon reasonable request.
